# A practice-based randomized controlled trial to improve medication adherence among Latinos with hypertension: study protocol for a randomized controlled trial

**DOI:** 10.1186/s13063-015-0815-x

**Published:** 2015-07-02

**Authors:** Antoinette Schoenthaler, Franzenith De La Calle, Miguel Barrios-Barrios, Aury Garcia, Maria Pitaro, Audrey Lum, Milagros Rosal

**Affiliations:** Department of Population Health, Center for Healthful Behavior Change, New York University School of Medicine, 227 East 30th Street, 634, New York, NY 10016 USA; Union Health Center, New York, NY 10001 USA; Division of Preventive and Behavioral Medicine, Department of Medicine, University of Massachusetts Medical School, 55 Lake Avenue North, Worcester, MA 01655 USA

**Keywords:** Medication adherence, Hypertension, Latinos, Health coach

## Abstract

**Background:**

Latinos experience disproportionately higher rates of uncontrolled hypertension as compared to Blacks and Whites. While poor adherence is a major contributor to disparities in blood pressure control, data in Latino patients are scant. More importantly, translation of interventions to improve medication adherence in community-based primary care practices, where the majority of Latino patients receive their care is non-existent.

**Methods:**

Using a randomized controlled design, this study evaluates the effectiveness of a culturally tailored, practice-based intervention compared to usual care on medication adherence, among 148 Latino patients with uncontrolled hypertension who are non-adherent to their antihypertensive medications. Bilingual medical assistants trained as Health Coaches deliver the intervention using an electronic medical record system-embedded adherence script. Patients randomized to the intervention group receive patient-centered counseling with a Health Coach to develop individualized self-monitoring strategies to overcome barriers and improve adherence behaviors. Health Coach sessions are held biweekly for the first 3 months (6 sessions total) and then monthly for the remaining 3 months (3 sessions total). Patients randomized to the usual care group receive standard hypertension treatment recommendations as determined by their primary care providers. The primary outcome is the rate of medication adherence at 6 months. The secondary outcome is reduction in systolic and diastolic blood pressure at 6 months.

**Discussion:**

If successful, findings from this study will provide salient information on the translation of culturallytailored, evidence-based interventions targeted at medication adherence and blood pressure control into practice-based settings for this high-risk population.

**Trial registration:**

NCT01643473 on 16 July 2012.

## Background

Latinos are the fastest growing ethnic group of the United States (US) and account for more than half of the total increase in the country’s population over the past decade [1]. This growth has been accompanied by a significant increase in cardiovascular disease (CVD)-related morbidity and mortality [2]. Despite increasing trends in the awareness and treatment of hypertension (HTN) among all groups, Latinos have the lowest blood pressure (BP) control rates in the US [3]. Although barriers to optimal HTN control, such as poor access and low awareness, have been used to explain the disparities in BP control between Latinos and Whites, BP control rates remain lower among Latinos who receive treatment compared to Whites [4]. This enigma may be explained by the disproportionately poorer adherence to prescribed antihypertensive medications among Latinos compared to Whites [5, 6].

Poor medication adherence is a major contributor to inadequate BP control, and is associated with 125,000 deaths annually [7]. While many interventions address poor adherence behaviors in hypertensive patients [8, 9], data in Latino patients are scant. More importantly, translation of adherence interventions to community-based primary care practices, where the majority of Latino patients receive care, is non-existent. Thus, the development of tailored interventions that target improving medication adherence in this high-risk population is needed in order to address the racial disparities in BP control between Latinos and Whites.

The Ayudando Latinos Hipertensos Para Mejorar Adherencia a los Medicamentos (ALMA) trial addresses this gap in the literature by evaluating the effect of a culturally tailored, practice-based intervention on medication adherence in 148 Latino patients with uncontrolled HTN, who are non-adherent to their antihypertensive medications and followed in a medical clinic in New York City. To facilitate translation into routine practice, the intervention is embedded into the clinic’s electronic medical record (EMR) system, and bilingual medical assistants (MAs) who are trained as Health Coaches deliver the patient-centered counseling.

### Study aims

The primary aim of the ALMA trial is to evaluate the effect of a culturally tailored, evidence-based adherence intervention (AI), delivered by bilingual Health Coaches versus usual care (UC), on medication adherence at 6 months among 148 Latino patients with uncontrolled HTN, who are non-adherent to their anti-hypertensive medications. The secondary aim is to evaluate the effect of the AI versus UC on BP reduction at 6 months. We hypothesize that the AI group will have a higher proportion of patients who are adherent to their antihypertensive medication; and a greater reduction in systolic BP (SBP) and diastolic BP (DBP) at 6 months as compared to the UC group. ALMA is one of the first trials to evaluate the effect of a culturally tailored, practice-based AI for Latinos with uncontrolled HTN. Moreover, trained MAs who assume the role of a Health Coach, and who often are the frontline healthcare workers in primary care practices, deliver the intervention; thus maximizing the likelihood of translating the study into clinical practice.

## Methods

### Study design

**Fig. 1:**
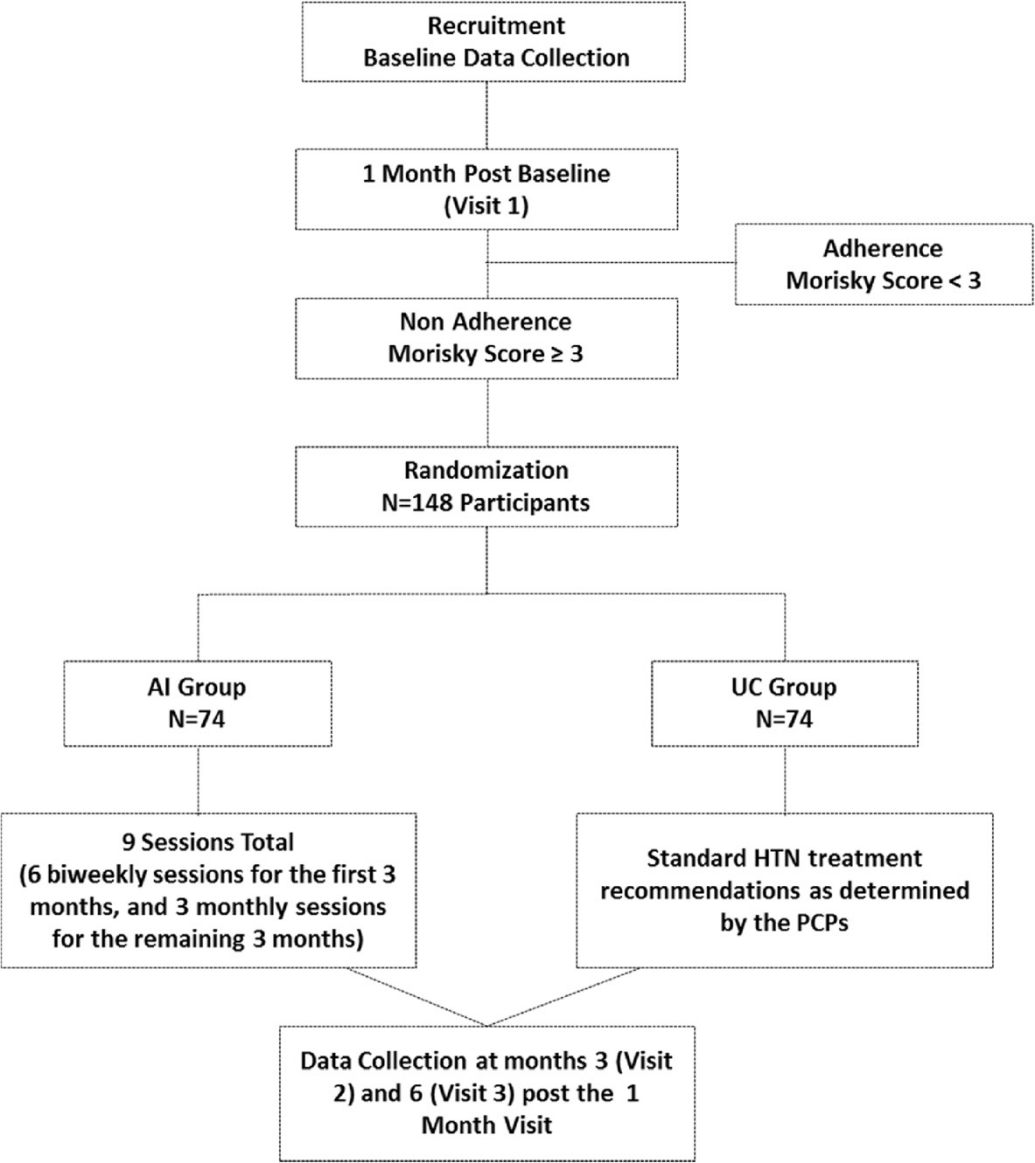
Flow of participants through the study

As depicted in Fig. 1, ALMA is a randomized controlled trial with 2 arms: a culturally tailored, practice-based AI arm, and a UC arm. Approximately 148 Latino patients with uncontrolled HTN and who are non-adherent to their antihypertensive medications are randomly assigned equally to either the AI or UC conditions. Patients randomized to the UC group receive standard HTN treatment recommendations as determined by their primary care providers (PCPs). Patients randomized to the AI group participate in 6 biweekly sessions with bilingual Health Coaches for the first 3 months and then 3 monthly sessions for the remaining 3 months (9 sessions total). Health Coaches utilize a culturallytailored adherence script to identify patient’s specific medication adherence barriers and to explore medication adherence facilitators. Based on the patients’ responses, the Health Coach then engages in targeted patient-centered counseling to assist patients in developing individualized self-monitoring strategies to overcome these barriers and improve adherence behaviors. Brief motivational interviewing (MINT) strategies form the basis of the adherence counseling. MINT is a patient-centered counseling approach that encourages patients to express their concerns about, and barriers to, taking medications, connect their personal values and goals to their health behaviors, enhance their motivation and confidence for change, and make a commitment to change [10]. We have previously demonstrated the effectiveness of using brief MINT to improve medication adherence in Black hypertensive patients followed in community-based practices [11].

Study assessments are conducted at baseline, 1 month post-baseline (visit 1), 3 months post the 1-month assessment (visit 2), and 6 months post the 1-month assessment (visit 3). The primary and secondary outcomes are assessed at the 3-month (visit 2) and 6-month (visit 3) follow-up visit. Patients receive US$5 at baseline, US$10 at the 1-month visit, US$10 at the 3-month visit, and US$10 at the completion of the 6-month visit (total of US$35 over the 6-month study).

### Study setting and patients

This study is conducted at a community-based medical clinic that serves predominantly Latino patients in New York City. The clinic employs 12 bilingual primary care providers, and 75 bilingual support and administrative staff (including 28 MAs and 7 Health Coaches). An Academic Community Advisory (ACA) Board comprised of four members of the clinic, each representing a key stakeholder group (i.e., patients, clinic staff, and clinic administrators), provides input throughout the study on recruitment, delivery of the intervention and on how best to retain patients.

The target enrollment for the study is 148 patients who meet the following study eligibility criteria: a) have uncontrolled HTN defined as BP > 140/90 mmHg on at least 2 consecutive visits in the past year of receiving care at the clinic (or BP > 130/80 mmHg for those with diabetes or kidney disease) and at least 1 CVD risk factor including hyperlipidemia or diabetes; b) take at least 1 antihypertensive medication; and c) self-identify as Latino and be ≥ 18 years of age. Patients are excluded if they: a) refuse or are unable to provide informed consent; b) currently participate in another HTN study; or c) have significant psychiatric comorbidity. The study protocol was reviewed and approved by the Institutional Review Board of New York University Langone Medical Center. All patients provide written informed consent to participate. The study is registered at www.clinicaltrials.gov: NCT01643473.

### Recruitment

Potentially eligible patients are identified via three methods. First, PCPs and their MAs refer patients who meet the eligibility criteria to a research assistant (RA). Referrals are done via EMR flags, or by calling the RA. The RA then conducts an onsite screening and consent visit with the referred patients. Second, the RA reviews the EMR using the *International Classification of Diseases* (ICD)-9 codes for the diagnosis of HTN (401–401.9). The PCPs of patients are notified of their potential eligibility and asked permission to enroll their patients into the study. Upon obtaining PCP consent, the RAs note the eligible patients’ appointment dates and approach the patients at their next clinic visit for screening and consent. Finally, flyers are also hung in the clinic waiting room.

### Randomization

After completion of the 1-month (post-baseline) visit, patients who are non-adherent to their prescribed antihypertensive medication (defined as having a Morisky Score ≥ 3) are randomly assigned to either the AI or UC group by the study statistician. Block randomization is used to ensure a roughly equal assignment of patients to the two groups. A block size of 8 or 16 varies randomly across the trial, with the investigators blind to the block size. All randomized subjects will be included in the analyses using intent-to-treat strategies [12]. Following Consolidated Standards of Reporting Trials (CONSORT) guidelines [13, 14], the randomization group names are kept in opaque envelopes in a locked cabinet away from the study site. Once a month, the study coordinator opens the envelopes to reveal patients’ group assignment. A RA informs patients of their group assignment by phone; at which time, they also discuss the telephone counseling schedule, if randomized to the AI arm and answers any additional questions. As is true for most behavioral interventions, neither the patient nor the interventionists (Health Coaches) or study staff can be blinded to the group assignment. To mitigate the potential for bias for the primary and secondary outcomes, an electronic monitoring device (EMD) is used to assess medication adherence and a validated automated BP device is used to assess BP.

### Description of the intervention

#### Development of the Adherence Intervention (AI)

The Common Sense Model of Self-regulation (CSM) is the theoretical framework underlying the AI. The CSM incorporates patients’ illness beliefs about the cause, symptoms, consequences, controllability, and timeline (acute versus chronic) of their condition into the conceptualization of adherence behaviors [15]. Moreover, given the dynamic nature of medication-taking behaviors over time, the five CSM illness beliefs are further categorized using Vrijens et al. medications adherence taxonomy [16, 17] into: initiation, implementation, and discontinuation of the recommended treatment plan. Initiation refers to patients’ willingness to take their medications as well as the perceived benefits and risks of the medications. Implementation is a continuous process that assesses how well patients’ daily medication-taking behaviors correspond to the prescribed regimen. Finally, discontinuation refers to early termination of the medication regimen by the patient.

These conceptual frameworks, in combination with a review of the published literature that examines the role of patients’ illness beliefs on adherence behaviors among Latinos with chronic diseases (i.e., diabetes, HIV), and preliminary results from our previous studies, formed the initial basis of the illness beliefs and adherence barriers addressed by the AI.

A formative phase was conducted during the first 8 months of the study, prior to the initiation of the trial and subject recruitment, to further refine the AI. Specifically, data from the focus groups and cognitive patient interviews, as well as feedback from the ACA Board were obtained to gain an accurate understanding of the multiple determinants of medication adherence (i.e., cultural, cognitive, psychosocial, behavioral, logistical) directly from the target patient population as well as the key stakeholders that deliver services to them. Overall, the findings showed that Latino patients form their beliefs about HTN and HTN medications with information received from family members, media, and to a lesser extent, physicians. Moreover, in spite of the fact that HTN was perceived as a treacherous and unpredictable disease, and medications were deemed an essential method to avoid adverse consequences, the need for medications was determined by the presence of perceived symptoms. Symptom alleviation was used as a marker for treatment efficacy and HTN-related risk reduction. Based on the focus group findings, a rubric for adherence counseling was developed to assist the Health Coaches in delivering the intervention. Specifically, the AI script encompasses a set of guidelines for counseling patients based on changes in their adherence behaviors overtime (i.e., improving adherence, or no change/worse adherence) and barriers and facilitators to medication adherence informed by findings from the focus groups.

To ensure consistency in the delivery of the intervention across English-speaking and Spanish-speaking Latino patients, the AI was further translated into the Spanish language by a professional translation service. Three semi-structured interviews were then conducted with bilingual Latino patients to garner feedback on the clarity of the English and Spanish versions of the AI, and to elicit additional culture-specific norms regarding initiation and implementation of the medication regimen, barriers to adherence, and specific strategies for developing behavioral action plans for taking medications as prescribed. The interviews lasted approximately 30 minutes and patients receive US$15 for their time. Finally, the ACA Board provided feedback on the cultural appropriateness of the AI and offered insight into the logistical and administrative barriers that can impede adherence in this population. Together, these data were used to further refine the AI to match the literacy and cultural needs of the Latino patient population at the clinic, and to inform important intervention messages during the delivery of the counseling sessions.

#### Integration of the finalized AI into the EMR

**Fig. 2:**
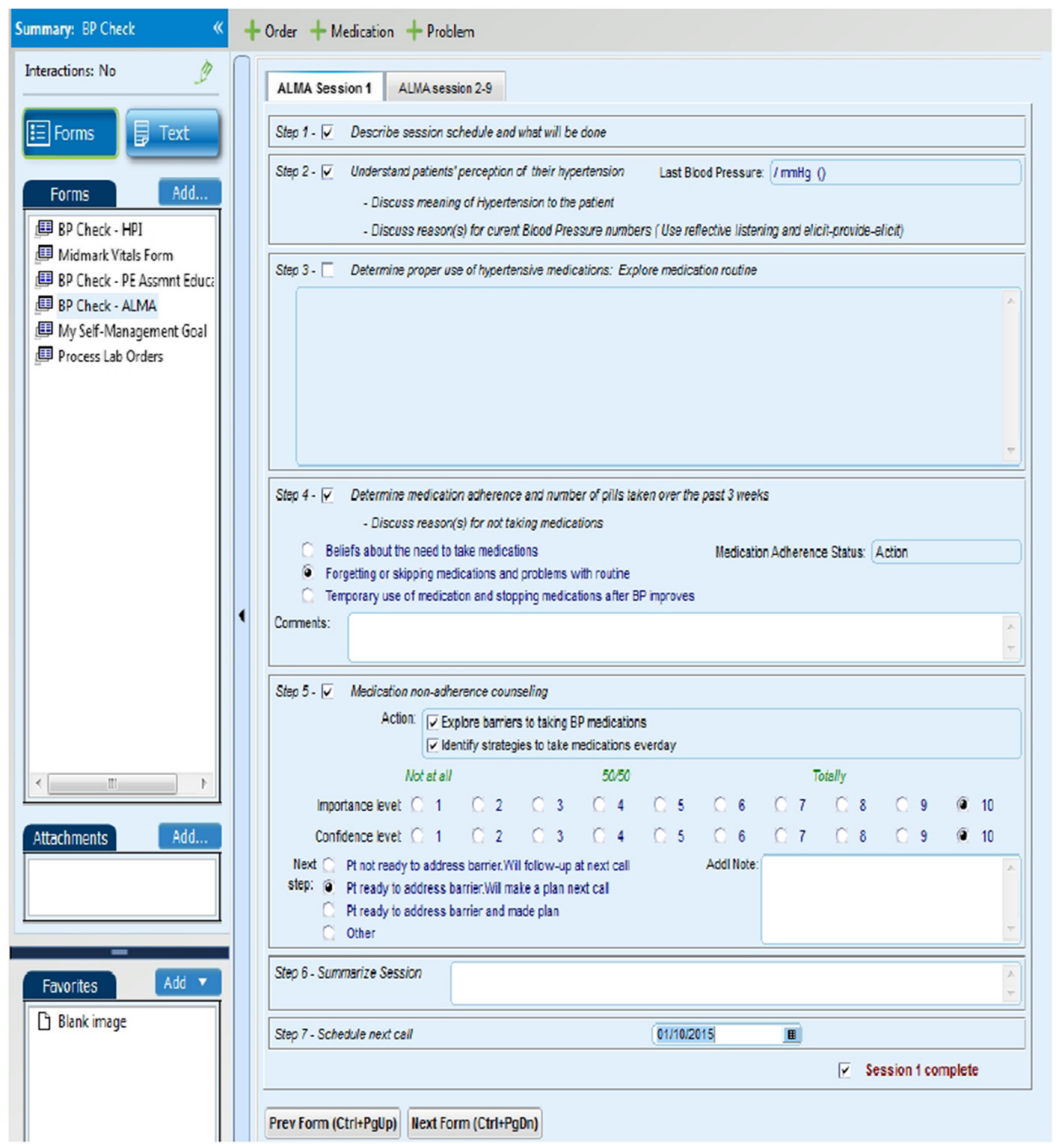
Example of Electronic Medical Record (EMR)-embedded adherence intervention template

The final AI was customized for the clinic’s EMR-system using a format similar to the clinic’s current educations materials and EMR templates (Fig. 2). This allows for standardization of procedures and tracking of patient progress. In addition, the EMR-embedded AI allows Health Coaches to systematically record patients’ responses and retrieve information and progress from previous sessions. It also includes tips for using MINT strategies to address patient barriers such as eliciting and responding to patients’ understanding of the causes, complications and treatment of HTN; perceived barriers to taking medications; and strategies for adoption of adherence behaviors. Thus, based on the patient’s responses, the Health Coach can engage in targeted patient-centered counseling to assist patients in developing individualized self-monitoring strategies to overcome barriers and improve adherence behaviors. Customization of the AI into the EMR also facilitates the integration of the intervention into the clinic workflow once the study has ended. Access to the AI is via a password-protected portal.

In order to minimize contamination of the UC group, several quality control measures were embedded in the EMRs. Upon randomization, patients’ EMRs are marked according to their group assignment. Health Coaches are not able to access the EMR adherence script for patients randomized to the UC arm, thereby preventing the utilization of the intervention with these patients. Moreover, Health Coaches are required by the clinic to document everything discussed in their sessions; thus, we will be able to review their notes to determine whether medication adherence was discussed with patients in the UC group and to what extent. As a secondary measure, at follow-up visits, contamination is measured based on patients’: 1) awareness of both study arms, 2) direct exposure to the intervention activities among those in the control arm, and 3) the nature of any contact and interaction with patients in the intervention arm.

#### Health coach training

As part of the standard of care at the participating clinic, all MAs undergo training in chronic disease management, development of effective communication skills, and principles of self-management and behavior change. The trainings include didactic and interactive sessions, direct observation, and skills assessment. Upon completion of the training, the MAs are promoted to Health Coaches, at which point they can deliver individual sessions with patients. A Health Coach’s typical clinic responsibilities include reviewing the daily panel of patients with the PCP in a morning huddle; scheduling appointments for telephone follow-up of test results; providing general health education; communicating to the patient, recommendations for treatment changes made by the PCP; seeing patients for BP checks and, if needed, referring the patient to the PCP for medication adjustment. Since patient visits with Health Coaches are standard of care at the clinic, regardless of group assignment; all patients have contact with a Health Coach.

For the purpose of this study, four of the seven Health Coaches employed at the clinic received additional training in the delivery of the tailored AI and patient-centered counseling techniques 1 month prior to initiation of the intervention. The 3-day interactive training included an overview of the project goals and study overview; an introduction to the facilitators and barriers of medication adherence in Latinos; and overview of self-monitoring strategies (i.e., use of reminder systems) to improve adherence. In addition, Health Coaches received an introduction to the basic principles of MINT techniques and problem-solving strategies [10] and also had the opportunity to practice the MINT strategies that form the basis of the intervention through role-plays and self-modeling with feedback from members of the MINT International Network of Trainers. Finally, the Health Coaches received an in-depth education on the content of the AI materials and practice sessions on the use of the EMR AI template and how to develop action plans through structured goal setting.

#### Adherence Intervention (AI) contacts

Patients randomized to the AI group participate in 9 sessions (6 biweekly sessions for the first 3 months, and then 3 monthly sessions for the remaining 3 months) with the trained bilingual Health Coaches who utilize the culturally tailored AI to identify patient’s specific medication adherence barriers and facilitators. Patient’s patterns of non-adherence, which are assessed during the baseline and 1-month study visit are also provided to the Health Coaches prior to the start of the intervention. Each counseling session lasts approximately 15 minutes and is conducted via telephone or in-person.

### Usual Care (UC) condition

Patients randomized to the UC group receive standard HTN treatment recommendations as determined by their PCP as well as the standard health coaching procedures followed at the clinic.

### Outcomes assessments

Study assessments for primary and secondary outcomes are completed at baseline, 3 months (post the 1-month visit), and 6 months (post the 1-month visit). All assessments are performed by trained RAs and include: (1) objective measures; (2) physiological measures, (3) self-report measures, and (4) EMR chart data. Table 1 summarizes the measures according to their timeline.

**Table 1:** Study measures by modality

Measures	Baseline	1 Month	3 Months	6 Months
Outcome measures				
Blood pressure measurements	X		X	X
Medication adherence (Electronic Monitoring Device (EMD))		X	X	X
Self-report measures				
Participant demographics	X			
Cognitive functioning (CARE-DIAG)	X			
Health literacy	X			
Medical comorbidity (Charlson Comorbidity Index)		X		X
Self-efficacy scale (MASE)		X	X	X
Intrinsic motivation (TSRQ)		X	X	X
Illness beliefs (IPQ-R)	X		X	X
Medication adherence (Morisky eight-item)	X		X	X
Attitudes about medication (Beliefs about Medication Questionnaire)	X		X	X
EMD diary		X	X	
EMD survey		X		X
PCAS survey		X		X
Electronic medical record data				
Chart review	X			X

*CARE-DIAG* Comprehensive Assessment and Referral Evaluation Dementia Diagnostic Scale, *IPQ-R* Illness Perceptions Questionnaire-revised, *MASE* Medication Adherence Self-Efficacy Scale, *PCAS* Primary Care Assessment Survey, *TSRQ* Treatment Self-Regulation Questionnaire

### Primary outcome

#### Medication adherence

The primary outcome is the rate of medication adherence at the 6-month study visit, assessed using electronic drug monitoring devices (EMD; Information Mediary Co.). EMDs are designed as pill bottles with an electronic chip in the cap that records a temporal history of the date, time, and interval between each dosing, thereby allowing for real-time tracking of adherence behaviors. Medication adherence rates by the EMD are calculated as the percent of prescribed doses removed by the patient during the study monitoring period using the formula:

$$ \mathrm{Number}\ \mathrm{of}\ \mathrm{doses}\ \mathrm{removed}/\mathrm{Number}\ \mathrm{of}\ \mathrm{doses}\ \mathrm{prescribed}\times 100 $$

In essence, this metric (known as taking adherence), is the proportion of days on which the patient took his or her medication as prescribed, divided by the total number of days that he or she is expected to take them (number of days in the assessed time period).

Detailed records of all patient emergency room visits and hospitalizations are kept to avoid erroneously penalizing patients for missing values for days during the study when their EMD is not in use. Such days are removed from the denominator of the formula in estimating the adherence rates for those patients. To control for the occurrence of “pocket dosing” (i.e., use of pill boxes, removing doses for travel), patients are asked to keep diaries of such periods, which will be accounted for in the analyses [18]. As in other trials, an EMD will be given to each patient to track the one antihypertensive medication taken most frequently and instructed on its use (without being told the primary purpose) [11, 19]. Although this does not reflect overall adherence rates, there is evidence that the pattern of adherence to one antihypertensive medication often reflects adherence to others [20]. In the event that patients are prescribed multiple medications, their PCP will be asked to identify the primary medication to be placed in the bottle.

In addition, medication adherence to prescribed antihypertensive medications is assessed with the well-validated eight-item self-report scale developed by Morisky that specifically addresses adherence to a prescribed mediation regimen [21]. The Morisky Adherence Scale is used to screen patients for their non-adherence during the first month of the study. Patients who are non-adherent at the 1-month study visit (score > 3) are considered eligible to participate. The measure has acceptable reliability (α = 0.83), correlates well with the previously validated 4-item version [36] (*r* = 0.64, *p* < 0.05) and was found to be able to correctly categorize BP control status in 80 % of cases [22].

### Secondary outcome

#### Blood pressure

The secondary outcome is the within-patient change in SBP and DBP from baseline to 6 months. BP is assessed using a well-validated automated device (WatchBP Office Device; Microlife; Golden, CO, USA) at all study visits, by trained RAs who take a series of BP readings after the patient has been seated for 5 minutes following American Heart Association guidelines. An average of three BP readings are used for each visit.

### Self-report measures

#### Cognition

Cognition is assessed using the Comprehensive Assessment and Referral Evaluation Dementia Diagnostic Scale (CDIAG) [23, 24]. This instrument was selected because it has been found to perform in a culture-fair manner [25] with better specificity among ethnically diverse groups, in comparison with other cognitive measures.

#### Patient demographic characteristics

The demographic data includes date of birth, place of birth, years in the US, primary language spoken as a child, primary language currently spoken at home, at work, and with friends and family, ethnicity, gender, household income, education level, marital status, religious affiliation, employment status, family finances, sources of income, financial strain, health insurance status, smoking status and alcohol use.

#### Comorbidity

Comorbid medical conditions are assessed with the Charlson Comorbidity Index (CCI) [26]. The CCI is a weighted index for prospectively classifying comorbid conditions, which takes into account the number and seriousness of comorbid diseases.

#### Illness beliefs

Illness beliefs are assessed with the Illness Perceptions Questionnaire-revised (IPQ-R) for HTN [27]. The IPQ-R is designed to assess the 5 illness beliefs and 6 emotional reactions to HTN based on the CSM, on a 5-point Likert type scale from strongly disagree to strongly agree (α range: 0.79 to 0.89). The IPQ-R has demonstrated good test-retest reliability and good predictive validity in patients with chronic disease [28].

#### Health literacy

Health literacy is assessed with the 36-item short-form Test of Functional Health Literacy in Adults (s-TOFHLA)-Spanish [29]. The s-TOFHLA is a reading comprehension test that has been linked to glycemic control in Spanish-speaking and English-speaking Latino patients with type 2 diabetes [30].

#### Self-efficacy

Self-efficacy is assessed with the Medication Adherence Self-Efficacy Scale (MASES; α = 0.95) [31]. Patients are asked to rate their confidence in taking their antihypertensive medications under a variety of situations that may pose difficulties. Higher scores reflect high self-efficacy.

#### Intrinsic motivation

Intrinsic motivation is assessed with the 14-item Treatment Self-Regulation Questionnaire (TSRQ) [32]. The TSRQ has 2 subscales: autonomous motivation and controlled motivation (α = 0.86 for each scale).

#### Beliefs about Medication Questionnaire (BMQ)

The BMQ was designed to assess the patient’s personal reasons for taking their medications [33]. Questions ask patients to rate how much they agree or disagree about statements that reflect their personal views about their medicines prescribed. Responses are given on a 5-point Likert scale (range: strongly agree to strongly disagree).

#### The Primary Care Assessment Survey (PCAS)

The PCAS is a 51-item patient-completed questionnaire designed to operationalize formal definitions of primary care including the definition posted by the Institute of Medicine Committee on the Future of Primary Care [34]. The survey measures 7 defining characteristics of primary care through 11 summary scales, including detailed measurement of the doctor-patient relationship (communication quality, patient trust, physician knowledge of patient, interpersonal treatment, relationship duration).

#### Electronic Monitoring Device (EMD) survey

Patient’s experience with the EMD will be assessed with a 17-item questionnaire from our previous studies [18]. The first 11 questions ask about patient’s use and comfort with the bottle. Sample items include: “I felt comfortable traveling with the pill bottle” and “I used the pill bottle every day.” Responses are given on a five-point Likert-type scale ranging from strongly agree to strongly disagree. The remaining six open-ended questions ask patients about the use of a pillbox, the number of medications they lay out, and to report any problems with the bottle.

### Chart extraction data

The study staff reviews all patients’ medical records at baseline and at 6months. Chart data extraction includes include duration of HTN, total number and classes of prescribed BP medications, as well as their doses and frequencies of ingestion, changes in dosages of BP medications, frequency of clinic visits; the use of other medications known to affect BP such as NSAIDS and hormone replacement therapy, medical comorbidity and clinic BP readings.

## Analysis

The power estimates are based on the primary aim using the basic comparisons of number (proportion) adherent in each group as the effect size. The nominal alpha value is set for a 2-sided test at the α = 0.05 level. Estimates of the hypothesized proportion of patients’ adherent in the AI versus UC group at the final follow-up are derived from values in the study by Lai et al. [35], the only comparable study with a Latino sample. Given the high proportion of adherent patients in the treatment group from that study, more conservative effect size estimates were used for the proposed study. Thus, the projected sample size is 148 patients (74 patients per group), which will provide 80 % power to detect a 0.20 difference in the adherence rate for patients in the AI versus UC groups. All analyses will be performed under an intent-to-treat design; therefore, all patients, including those who drop out of the study, will be invited back for the final assessments.

While randomization is expected to produce well-balanced groups, analyses will be done to determine any baseline differences between the groups on demographic or prognostic variables, using chi-square analyses for categorical variables and analysis of variance (ANOVA) for continuous ones. If significant differences are found or there is scientific plausibility of an association with the outcome variable, these variables will be included in a logit model to predict group assignment. Data from the initial group comparisons on patient characteristics (including the baseline primary outcome and other patient characteristics) will also allow for the estimation of a logit model of dropout. If the results indicate that attrition is significantly related to one or more baseline characteristic, predicted values from the final logit model will be used as a covariate in all subsequent analyses, thereby controlling for differential attrition.

### Analyses for the primary aim

The primary analysis will be a 2 (group) × 2 (adherence status) chi-square test of independence. The expectation is that the randomization of patients to treatment arm and the absence of significant selection and/or attrition biases will obviate the need for any covariates in the analysis. However, in the unlikely event that imbalances are identified and covariates are added to the model, a generalized linear model (logistic regression) will be used. In addition to two-sided tests of significance, the effects will be estimated with odds ratios.

### Analyses for the secondary aim

BP will be treated as continuous SBP and DBP variables and the effect of treatment on these variables will be assessed using linear mixed effects regression models, with time coded based on the WatchBP date stamp and treatment dummy coded as 0 = UC and 1 = AI. The critical test will be the time × treatment interaction. Time will be treated as a random effect. Mixed effects regression models have several advantages over traditional repeated measures multivariate analysis of variance (MANOVA) models, including the ability to include all patients in the analysis rather than only those with complete data. The parameter estimates from the regression models will be reported and used to calculate predicted scores to describe the effect of the AI on these outcomes.

### Exploratory analyses

Data on the self-report measures of self-efficacy, intrinsic motivation, beliefs about medications, and illness beliefs at 3 time points will allow for the examination of these variables as possible mediators on the outcomes at 6 months. Moderating effects of acculturation and health literacy will also be evaluated. These data may contribute to understanding why the proposed intervention worked.

## Discussion

### Study implementation: challenges and lessons learned

We have encountered several “real world” barriers to implementation of the ALMA study protocol as originally conceived. Several of the challenges we faced relate to the difficulties involved moving away from an intervention delivered by research staff to that of clinic staff with competing priorities. This implementation model adds a new level of complexity as researchers must contend with the need to uphold the methodological rigor of a randomized clinical trial while maintaining the flexibility to work with staff within a busy medical practice. The two main barriers to date are patient recruitment and delivery of the intervention during times that fit both patients’ and Health Coaches’ schedules. Below, we summarize the strategies we have employed to address these challenges as a resource for other researchers who are interested in utilizing indigenous clinic staff for intervention delivery in community-based practices.

### Recruitment challenges

Upon initiation of the trial, we experienced several challenges in patient recruitment. For example, we encountered a higher than expected rate of patient refusal for study participation. The most common reasons for declining participation include long work schedules, inability to receive study calls while at work, competing priorities (i.e., being too sick, caring for a sick person), patient travel to their home country for long periods of time, and overall lack of interest in participating. Recruitment was also prolonged during the first months of the study due to technical difficulties with the wireless EMDs that were originally selected to assess patient adherence. The main barrier to using the wireless EMDs was the frequent disruptions between the device and its communication hub, which led to transferring incomplete data to the research portal. In addition, some patients expressed difficulty in understanding how to use the device, were dissatisfied with its use, and/or used the device incorrectly. The combination of these factors paired with a higher than expected rate of physician turnover at the clinic has prolonged patient recruitment and reduced the overall pool of potentially eligible patients for recruitment.

In order to mitigate these recruitment challenges, we made several refinements to the study protocol. First, the PCPs and their MAs select patients from a list of potentially eligible patients identified from a HTN registry. Once the PCPs and their MAs select patients for the study, the patients’ appointment dates are noted, and an introductory study postcard is sent to inform patients of the study. Generally, this has increased patients’ receptiveness to talk with the RA during their clinical visit because it eliminates the element of surprise by giving patients prior notice before being approached. It also helps to decrease patients’ skepticism about the study because the postcard is endorsed by their PCP, thereby signifying an affiliation with the clinic. Second, for patients identified via EMR searches, an ALMA sticker label is placed on the patients’ huddle sheet to remind PCPs and MAs of patients’ eligibility for the study. The PCPs and/or MA can then briefly introduce the study to eligible patients during the clinic encounter. Third, once a patient completes the registration process, the RA coordinates with the MA to identify the most appropriate point during the visit to meet with the patient and ascertain their interest in participating in the study. This ensures that the study staff is not disrupting the clinic workflow while trying to maximize their reach of eligible patients. To increase visibility, advertising materials are posted throughout the clinic such as placing flyers in waiting rooms; hanging large posters in exam rooms, and creating displays in the clinic’s health showcase. Finally, we elicit additional feedback from the clinic staff and leadership on how to best obtain patient referrals and recruit patients (i.e., attending the clinic’s staff and PCP meetings periodically; a sponsorship of a breakfast for clinic administrative staff) as well as hold ongoing meetings with the ACA Board. The wireless EMD was also changed to another model that, while not being wireless, has high acceptability and usage rates by the study population (EMD return rates are as follows: 90 % at the 1-month and 3-month visit and 92 % at the 6-month visit).

### Challenges with intervention delivery

During the formative phase, several modifications were made to the design of the AI template embedded in the EMR. First, based upon beta-testing with the Health Coaches and feedback from key stakeholders, the study team refined the design of the AI template to improve its usability and flow during the delivery of the counseling sessions. For example, interactive features were added such as command buttons to facilitate navigating through the AI template and accessing the built-in EMR goal-setting template, directly from the AI template. Additional checkboxes containing the adherence barriers and facilitators garnered from the focus groups were also added to minimize the amount of note-taking and facilitate data collection. Finally, a comment box was added to prevent loss of data that was not captured in the checkboxes. These modifications improved the AI templates ease of use as well as made key data from previous sessions easily accessible, which the Health Coaches use to familiarize themselves with patients’ most recent adherence behaviors.

We have also encountered several “real world” challenges to the delivery of the intervention. One of the most frequent challenges is patients’ limited time availability to receive the counseling calls. Patients who are employed often have a short window of availability (approximately 1–2 hours) during their work schedule to receive the calls, and most are only available before their workday begins or after their shift has ended; times during which the Health Coaches are not at work. This time barrier paired with the competing clinic duties of the Health Coaches poses the greatest challenge to the delivery of the intervention. For these reasons, the study protocol was modified to include having the study staff send periodic reminder flags to the Health Coaches (based upon Health Coaches’ request) regarding outstanding and/or upcoming counseling sessions as well as sending reminder postcards to patients they are unable to reach. Additional strategies to increase intervention attendance include: coordinating with the Health Coaches who work extended rotating hours in the morning and night (i.e., before 9:00 a.m., and after 5:00 p.m.) to prioritize calling efforts for those patients with limited availability; and increasing the amount of communication, as early as after the first call attempt, between the study staff and the Health Coaches if they are unable to reach a patient. This allows the study staff to reach patients with tight schedules beyond clinic hours and proactively arrange appointments with Health Coaches.

A second “real world” challenge relates to audiotaping the counseling sessions as a measure of treatment fidelity. While, all Health Coaches were provided with audio recorders and microphones to facilitate taping, they reported not being able to comply with the recordings due to time constraints and the need to have the equipment stored in a location that was not readily accessible, which hindered the recording process. As a partial solution to this challenge, Health Coaches receive numerous booster trainings to prevent skill decay as well as to maintain adherence to the study protocol. Moreover, the interactive AI template in the EMR facilitates collection of data on the content of each counseling session.

### Retention challenges

Due to the patient time constraints discussed above, retention strategies were also modified to facilitate the completion of study questionnaires either on-site or over the phone. For patients who work full-time, this decreased the time burden in terms of having to return to the clinic on days when they did not have a medical appointment scheduled, or if they did not have time to complete the visit on the day they were at the clinic. A brief in-person visit is scheduled for patients who complete visit surveys over the phone to take their BP. Moreover, the assessment protocol was modified by moving several of the questionnaires from the baseline visit to the 1-month study visit to prevent patient fatigue. The health literacy questionnaire was also moved to the baseline visit (from the 1-month visit) in order to facilitate the administration of the 1-month visit by phone.

Additional strategies implemented to increase retention include: (1) mailing appointment reminder postcards, and/or informational postcards for patients who are difficult to reach, or have no medical appointments within a study visit time window to schedule the visit; (2) updating patients’ contact information during each study visit, and, (3) regularly checking patients’ medical appointments to coordinate the study visits on the day that patients are already scheduled to come to the clinic. These changes have resulted in an overall average 87 % retention rate at the study follow-up visits.

## Trail status

The ALMA trial began in October 2012. To date, we have randomized a total of 112 patients, of which 54 are in the AI group and 58 are in the UC group. Patient recruitment is still ongoing.

## Abbreviations

ACA Board: Academic Community Advisory Board
AI: adherence intervention
ALMA: Ayudando Latinos Hipertensos Para Mejorar Adherencia a los Medicamentos
ANOVA: analysis of variance
BP: blood pressure
BMQ: Beliefs about Medicines Questionnaire
CCI: Charlson Comorbidity Index
CDIAG: Comprehensive Assessment and Referral Evaluation Dementia Diagnostic Scale
CONSORT: Consolidated Standards of Reporting Trials
CSM: Common Sense Model
CVD: cardiovascular disease
DBP: diastolic blood pressure
EMD: electronic monitoring device
EMR: electronic medical record
HTN: hypertension
ICD: International Classification of Diseases
IPQ-r: Illness Perception Questionnaire-revised
MA: medical assistant
MANOVA: multivariate analysis of variance
MASES: Medication Adherence Self-Efficacy Scale
MINT: motivational interviewing
NSAIDs: non-steroidal anti-inflammatory drugs
PCAS: Primary Care Assessment Survey
PCP: primary care provider
RA: research assistant
SBP: systolic blood pressure
s-TOFHLA: short-form Test of Functional Health Literacy Assessment
TSRQ: Treatment Self-Regulation Questionnaire
UC: usual care
US: United States
